# Shaping efficiency versus dentin conservation: a micro-CT taper-based analysis

**DOI:** 10.1007/s00784-026-06843-6

**Published:** 2026-04-01

**Authors:** Emmanuel João Nogueira Leal da Silva, Thiago Moreira Soares e Silva, Ana Flávia Almeida Barbosa, Graziela dos Santos Massa, Carolina Oliveira de Lima, Marco Aurélio Versiani

**Affiliations:** 1 Grande Rio University (UNIGRANRIO), Rio de Janeiro, RJ Brazil; 2 Department of Endodontics, Rio de Janeiro State University (UERJ), Rio de Janeiro, RJ Brazil; 3 Department of Endodontics, Juiz de Fora Federal University (UFJF), Governador Valadares, MG Brazil; 4 Oral Health Center, Brazilian Military Police, Belo Horizonte, Minas Gerais Brazil

**Keywords:** Dentin removal, Instrument taper, Mandibular molars, Micro-CT, Nickel-titanium

## Abstract

**Objectives:**

To evaluate the influence of instruments with identical tip diameters but increasing taper on shaping efficiency and dentin preservation in the root canals of mandibular molars using micro-CT.

**Materials and methods:**

Twelve extracted mandibular molars with Vertucci Type III mesial canals and Type I distal canals were scanned using micro-CT before and after each instrumentation step. Mesial canals were sequentially prepared with 25/0.03, 25/0.05, 25/0.06, and 25/.08v instruments, whereas distal canals were prepared with 40/0.03, 40/0.05, and 40/.06v instruments. Co-registered datasets were used to quantify canal volume, surface area, percentage of unprepared canal surface, dentin removal, and minimum dentin thickness. In addition, dentin thickness values were categorized according to clinically relevant thresholds (< 0.5 mm, 0.5–1.0 mm, and > 1.0 mm). Data were analyzed using generalized linear and mixed-effects models (α = 0.05).

**Results:**

In mesial canals, unprepared area decreased significantly with larger tapers; reductions were significant with 25/0.06 (–21.9%) and 25/.08v (–37.9%), but not with 25/0.05. Dentin removal increased markedly and progressively with taper, reaching ~ 350% with 25/.08v compared with 25/0.03 (*p* < 0.001). In distal canals, taper did not significantly affect the unprepared area; however, dentin removal increased significantly with 40/0.05 and 40/.06v. Directional analyses confirmed a stepwise increase in dentin reduction, following the pattern mesiobuccal > mesiolingual > distal at all levels. Threshold analysis showed that the proportion of dentin thickness measurements within the 0.5–1.0 mm range increased with larger tapers, particularly on the distal aspect of the mesial roots.

**Conclusions:**

Increasing instrument taper improves shaping efficiency in mesial canals but substantially increases dentin removal and shifts dentin thickness toward potentially vulnerable ranges. Conservative taper selection may therefore better balance shaping effectiveness and structural preservation in mandibular molars.

**Clinical Relevance:**

Larger tapers may improve canal wall contact but can substantially reduce dentin thickness in anatomically vulnerable regions, emphasizing the importance of anatomy-guided taper selection during root canal preparation.

## Introduction

Effective root canal preparation remains a critical determinant of endodontic treatment success, as it directly influences irrigation efficacy, microbial reduction, and the quality of obturation [1, 2]. The primary objectives of canal shaping include the creation of a continuously tapered preparation, preservation of the original canal anatomy, and adequate removal of infected dentin while maintaining the structural integrity of the root structure [3]. However, achieving a balance between shaping efficiency and dentin conservation continues to represent one of the greatest challenges in clinical endodontics, particularly in anatomically complex teeth such as mandibular molars.

Mandibular molars often present thin mesial roots with flattened or oval-shaped canals and irregularities such as isthmuses and fins, which contribute to a significant percentage of untouched canal walls even after mechanical preparation [4]. Previous micro-computed tomography (micro-CT) studies have demonstrated that a substantial proportion of canal surfaces may remain unprepared regardless of the instrumentation techniquerevious micro-computed tomography (micro-CT) studies have demonstrated that a substantial proportion of canal surfaces may remain unprepared regardless of the instrumentation technique [5–7], raising concerns about the effectiveness of mechanical debridement alone. In this context, increasing the taper of endodontic instruments has been suggested as a strategy to improve canal wall contact and reduce the percentage of unprepared areas, potentially enhancing irrigation dynamics and disinfection [8–10]. On the other hand, larger tapers have consistently been associated with increased dentin removal, particularly in danger zones such as the furcal aspect of mesial roots in mandibular molars [11–14]. Excessive dentin removal in these regions significantly compromises root strength and increases susceptibility to vertical root fracture [15, 16], one of the leading causes of endodontic failure. Consequently, the selection of instrument taper must be carefully balanced to optimize shaping effectiveness without jeopardizing the biomechanical integrity of the root.

Recent advancements in nickel-titanium (NiTi) technology have resulted in a wide range of instruments sharing the same apical diameter but exhibiting different taper designs. While this provides clinicians with greater flexibility in apical enlargement strategies, it also raises important questions concerning the true benefit and biological cost of increasing taper alone. Previous investigations have primarily focused on comparing different apical sizes or contrasting distinct instrumentation systems [17, 18]. However, there is a limited number of studies specifically evaluating the stepwise effect of increasing taper while maintaining a constant tip diameter, especially in both mesial and distal canals of mandibular molars using high-resolution micro-CT [8, 9, 19]. Therefore, the aim of the present study was to evaluate the influence of instruments with tip size 25 and progressively increasing tapers (0.03, 0.05, 0.06, and .08v) and tip size 40 and progressively increasing tapers (0.03, 0.05, and .06v) on the percentage of unprepared canal surface, volume of dentin removed, and changes in dentin thickness in the mesial and distal root canals of mandibular molars, respectively. The null hypothesis was that increasing the instrument taper would not significantly affect the extent of canal surface preparation, dentin removal, or remaining dentin thickness.

## Materials and methods

### Sample size calculation

Sample size was estimated at α = 0.05 and 80% power, based on the largest pilot differences in unprepared area (45.4% vs. 35.3%; effect size = 1.97) and dentin removal (0.6% vs. 2.6%; effect size = 3.0) in six mesial canals. The minimum required sample sizes were 10 and 6, respectively. To account for the potential loss of specimens during the experimental procedures, 12 teeth were therefore included in the final sample.

## Specimen selection

After local ethics committee approval (Protocol No. 74873021.4.0000.5283), twelve extracted human mandibular molars with fully formed apices, two separate roots, and radiographically visible canals, and without prior endodontic treatment, resorption, or fracture, were selected from a tooth bank. Specimens were scanned using a micro-CT device (SkyScan 1174; Bruker-microCT, Kontich, Belgium) at 50 kV, 800 µA, 22 μm pixel size, 180° rotation (0.7° steps), and a 0.5-mm aluminum filter. Reconstructions (NRecon v1.6.1.0; Bruker-microCT) were processed with beam hardening (35%), ring artefact correction (7), and smoothing (7). Canal configuration was assessed (CTVol v2.3.1; Bruker-microCT), and canal volume (mm³) and surface area (mm²) were quantified using CTAn v1.6.6.0 (Bruker-microCT). Only teeth with Vertucci Type I [20] distal canals and Type III [20] mesial canals with isthmus Type I [21] starting at the middle third were included. These pre-operative measurements were used to confirm baseline anatomical comparability among specimens and served as reference values for subsequent analyses. Teeth were stored in distilled water until use.

### Root canal preparation

After conventional access, apical patency was confirmed with sizes 08 and 10 K-files, and a glide path was established with a size 15 K-file to the working length (WL), set 1 mm short of the apical foramen. The mesial and distal roots were coated with a light-curing resin (Whitegold Protector Blue; Dentsply Sirona) to simulate a closed system and mounted in a dental mannequin.

The mesiobuccal (MB) and mesiolingual (ML) canals were prepared sequentially with 25/0.03, 25/0.05, and 25/0.06 rotary instruments (Bassi Logic, Bassi Endo, Ocoee, FL, USA; 600 rpm, 2–4 N·cm), followed by a 25/.08v reciprocating instrument (Reciproc R25; VDW, Munich, Germany). Distal canals were shaped with 40/0.03 and 40/0.05 rotary instruments (Bassi Logic; 600 rpm, 2–4 N·cm), followed by a 40/.06v reciprocating instrument (Reciproc R40; VDW). All instrumentation was performed using a VDW Silver motor, employing three 3-mm pecking motions per cycle until the working length was reached. Reciprocating instruments were operated in RECIPROC ALL mode, in accordance with the manufacturer’s recommendations. A single instrument was used for each tooth. Irrigation was carried out with 2.5% NaOCl, using a total volume of 10 mL per canal at each preparation step, delivered through a 30G needle positioned 2 mm short of the WL. Final irrigation comprised 3 mL of 2.5% NaOCl, 3 mL of 17% EDTA for 3 min, and a final 3 mL NaOCl rinse. All procedures were performed by a single experienced operator under ×16 magnification and rubber dam isolation. After each enlargement step, micro-CT scans were repeated using the same parameters as baseline.

### Image analysis

Postoperative 3D models of the roots and root canals were created (CTAn v1.6.6.0; Bruker-microCT) and co-registered with the corresponding preoperative datasets by means of affine registration in 3D Slicer v4.4.0 (http://www.slicer.org). After each enlargement step, quantitative analyses were performed to determine canal volume (mm³), surface area (mm²), the percentage of unprepared canal surface, and the volume of dentin removed (CTAn v1.6.6.0, Bruker-microCT). Unprepared areas were identified as static voxels, defined as those remaining unchanged before and after preparation [22]. These were expressed as a percentage of the total surface voxels using the formula: (SV_n_ × 100) / SV_t_, where SV_n_ represents static voxels and SV_t_ the total surface voxels. Dentin volume (mm³) and percentage dentin removal were calculated by subtracting the segmented root dentin volume before and after instrumentation. A color-mapped 3D model of dentin thickness (CTAn v1.6.6.0; Bruker-microCT) was qualitatively assessed before and after each preparation step (CTvox v3.3.1; Bruker-microCT). In addition, color-coded cross-sectional images were used to locate and measure the minimum dentin thickness at 1.0-mm intervals, beginning at the furcation level and extending 3 mm apically for each canal. Minimum dentin thickness measurements obtained in all evaluated level was categorized according to predefined thresholds (< 0.5 mm, 0.5–1.0 mm, and > 1.0 mm), and the frequency of observations within each category was calculated for the mesiobuccal, mesiolingual, and distal canals before preparation and after each instrumentation step in both mesial and distal aspects of the root. All image analyses were performed by a micro-CT expert who was blinded to the preparation protocols.

### Statistical analysis

Model assumptions were evaluated using distribution-based modelling and simulation-derived residual diagnostics rather than classical normality and homoscedasticity tests. Data distributions were assessed using Cullen-Frey plots and histograms to determine the appropriate error structure. Generalized linear models (GLMs) were used to evaluate the effect of instrument type on the percentage of unprepared canal surface and the volume of dentin removed in mesial and distal canals. In mesial canals, unprepared area was approximately normally distributed and analyzed with a Gaussian GLM (identity link), whereas dentin removal was right-skewed and heteroscedastic and was therefore modelled with a Gamma GLM (log link). In distal canals, both variables were positively skewed and analyzed using Gamma GLMs with log links. Instrument type was included as a fixed factor in all models. Dentin thickness reduction was analyzed using linear mixed-effects models, with instrument type as a fixed effect and sample identity as a random intercept to account for repeated measures. *Post hoc* comparisons were performed using Tukey adjustment, and results are presented as estimated marginal means with 95% confidence intervals. All analyses were conducted in R (version 4.x) using the stats, fitdistrplus, lme4, DHARMa, and emmeans packages with α = 0.05. Model diagnostics conducted using DHARMa residual analyses included tests for uniformity, dispersion, and outlier influence, to confirm adequate model fit.

## Results

**Fig. 1:**
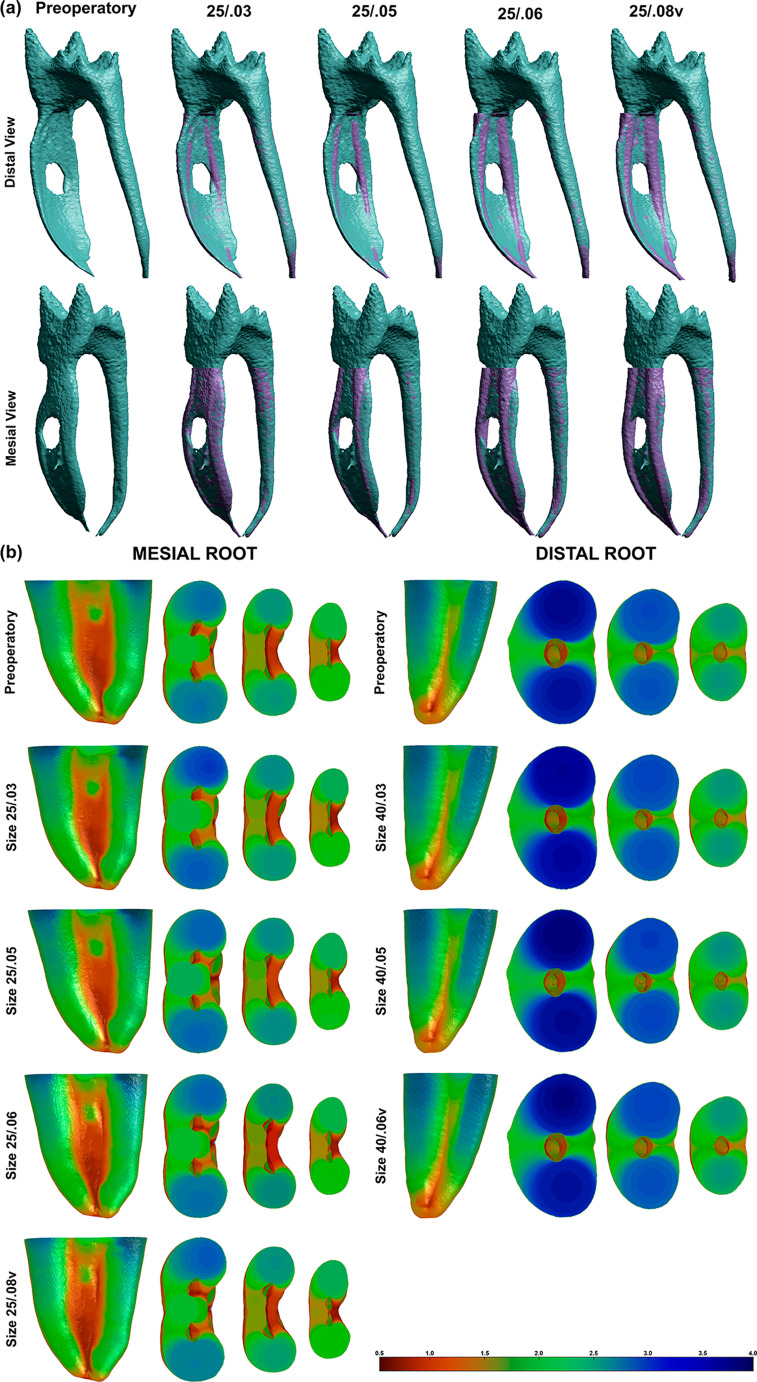
(**a**) Mesial and distal views of representative 3D models of a mandibular molar before and after root canal enlargement using rotary instruments with different tapers and same tip diameter; (**b**) Representative color-coded 3D models of the mesial (distal aspect) and distal (mesial aspect) roots of a mandibular molar before and after root canal enlargement with instruments with same tip and different tapers. Thick structures are represented in blue and green, while areas of thin dentine are highlighted in red

**Fig. 2:**
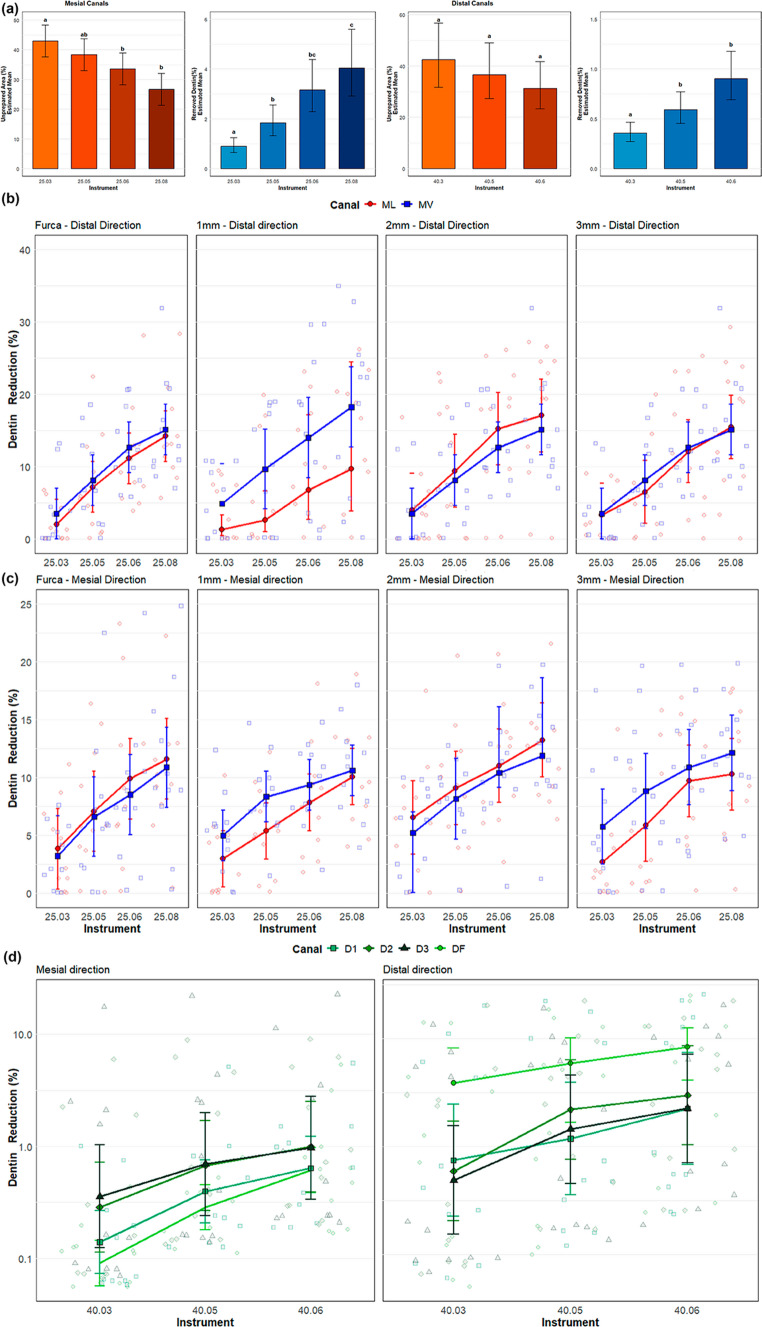
(**a**) Estimated means of dentine removed and unprepared area in mesial and distal canals after preparation with instruments of increasing taper. Mean dentin reduction (± SE) across sequential instruments in the mesial (**b**) and distal (**c**) directions for the MB, ML, and (**d**) distal canals. Error bars represent the standard error of the mean, and individual data points are displayed in light shading

In the mesial canals, increasing taper reduced the unprepared area (Fig. 1a; Table 1). Compared with 25/0.03, the reduction with 25/0.05 (− 10.8%) was not significant (β = −4.65, *p* = 0.22), whereas significant decreases were observed with 25/0.06 (− 21.9%; β = −9.39, *p* = 0.016) and 25/.08v (− 37.9%; β = −16.29, *p* < 0.001) (Fig. 2a; Table 1). Instrument type had a highly significant effect on dentin removal (*p* < 0.001), which increased by ~ 105% with 25/0.05 (β = 0.72, *p* = 0.003), ~ 252% with 25/0.06 (β = 1.26, *p* < 0.001), and ~ 350% with 25/.08v (β = 1.50, *p* < 0.001) relative to 25/0.03 (Fig. 2a; Table 1). In the distal canals, taper did not significantly influence the unprepared area (*p* > 0.05). The mean unprepared area for 40/0.03 was ~ 42.5%, with non-significant reductions for 40/0.05 (− 13.7%; β = −0.15, *p* = 0.47) and 40/.06v (− 26.4%; β = −0.31, *p* = 0.14). In contrast, dentin removal increased significantly with larger tapers, by ~ 66.2% with 40/0.05 (β = 0.51, *p* = 0.009) and ~ 154.0% with 40/.06v (β = 0.93, *p* < 0.001) (Fig. 2a; Table 1).

**Table 1:** Mean ± standard deviation of volume (mm³), surface area (mm²), unprepared canal walls (%), dentin removed (%) measured in the mesial and distal root canals of mandibular molars, before and after sequential root canal enlargement using instruments with different tapers. Different superscript letters indicate significant differences between root canal preparations with the same tip but different tapers (*p* < 0.05)

Root Canal	Preparation Step	Volume	Surface area	Unprepared Area	Dentin Removed
Mesial	Preoperatory	9.0 ± 3.6	65.7 ± 19.3	-	-
	25/0.03	9.5 ± 3.7	67.4 ± 19.4	43.4 ± 10.5^A^	0.5 ± 0.2^A^
	25/0.05	10.3 ± 3.8	68.6 ± 19.8	38.5 ± 10.2^AB^	1.0 ± 0.4^B^
	25/0.06	11.2 ± 3.9	70.5 ± 19.5	33.6 ± 7.8^B^	1.7 ± 0.9^BC^
	25/.08v	11.9 ± 3.7	72.1 ± 19.2	26.3 ± 9.6^B^	2.3 ± 0.9^C^
Distal	Preoperatory	9.9 ± 5.3	44.0 ± 14.1	-	-
	40/0.03	10.4 ± 5.4	45.6 ± 14.0	44.7 ± 17.5^A^	0.3 ± 0.2^A^
	40/0.05	10.6 ± 5.4	45.9 ± 14.0	38.5 ± 16.9^A^	0.6 ± 0.3^B^
	40/.06v	10.8 ± 5.4	46.5 ± 13.9	32.8 ± 17.7^A^	0.9 ± 0.4^B^

Directional analyses demonstrated a consistent, stepwise increase in dentin reduction with increasing taper across all canal types, with the magnitude following MB > ML > D from the furcation to the deepest levels (Figs. 1b and 2b-d). In the distal direction, reductions increased from 25/0.03 to 25/.08v, with significant differences in nearly all comparisons; only 25/0.05 versus 25/0.03 in the MB canal at 1 mm was non-significant (*p* = 0.067) (Figs. 1b and 2b). The ML canal showed significant increases from 25/0.06 onward at all depths, while in the distal canal the effect was smaller but still significant, beginning at 40/.06v in the furcal region and at 40/0.05 at 2–3 mm (Figs. 1b and 2b-d). A similar pattern was observed in the mesial direction, with highly significant increases in the MB canal and generally significant increases in the ML canal, apart from occasional non-significant differences with 25/0.05 at lower levels (Figs. 1b and 2c).

When dentin thickness values were categorized according to clinically relevant thresholds, a progressive shift toward thinner dentin walls was observed with increasing taper, particularly in the mesial canals (Table 2). In both the MB and ML canals, the number of measurements within the 0.5–1.0 mm range increased progressively with enlargement, particularly on the distal aspect, rising from 18.2% before preparation to 45.5% after the 25/.08v instrument in the MB canal, and from 45.5% preoperatively to 50% after the largest taper in the ML canal. In contrast, distal canals generally maintained greater dentin thickness, although a shift toward the 0.5–1.0 mm range was observed after preparation with larger tapers. No measurements below 0.5 mm were observed in the mesial canals, whereas only isolated occurrences were detected in the ML canal before preparation (Table 2).

**Table 2:** Distribution of dentin thickness measurements according to predefined thresholds (< 0.5 mm, 0.5–1.0 mm, and > 1.0 mm) in the mesiobuccal, mesiolingual, and distal canals at the furcation level before preparation and after each instrumentation step

		Mesiobuccal Canal					Mesiolingual Canal					Distal Canal			
Root Aspect	Dentin Thickness	Before Preparation	25/0.03	25/0.05	25/0.06	25/.08v	Before Preparation	25/0.03	25/0.05	25/0.06	25/.08v	Before Preparation	40/0.03	40/0.05	40/.06v
Mesial	< 0.5 mm	-	-	-	-	-	-	-	-	-	-	-	-	-	-
	0.5–1.0 mm	1 (2%)	1 (2%)	3 (7%)	3 (7%)	4 (9%)	2 (5%)	2 (5%)	4 (9%)	7 (16%)	8 (18%)	-	-	1 (2%)	1 (2%)
	> 1.0 mm	43 (98%)	43 (98%)	41 (93%)	41 (93%)	40 (91%)	42 (95%)	42 (95%)	40 (91%)	37 (84%)	36 (82%)	44 (100%)	44 (100%)	43 (98%)	43 (98%)
Distal	< 0.5 mm	-	-	-	-	1 (2.3%)	-	-	-	-	-	-	-	-	-
	0.5–1.0 mm	8 (18.2%)	12 (27.3%)	14 (31.8%)	20 (45.5%)	20 (45.5%)	11 (25%)	14 (32%)	18 (41%)	21 (48%)	22 (50%)	-	41 (93.2%)	37 (84.1%)	37 (84.1%)
	> 1.0 mm	36 (81.8%)	32 (72.7%)	30 (68.2%)	24 (54.5%)	23 (52.2%)	33 (75%)	30 (68%)	26 (59%)	23 (52%)	22 (50%)	44 (100%)	3 (6.8%)	7 (15.9%)	7 (15.9%)

Measurements were obtained at 1-mm intervals from the furcation level to 3 mm apically on both the mesial and distal aspects of the root canals. Values are expressed as absolute frequencies with percentages in parentheses

## Discussion

The present study provides stepwise evidence that increasing instrument taper, while keeping the apical diameter constant, affects both shaping effectiveness and dentin preservation in mandibular molars. Overall, the findings show that taper is a key factor in determining canal wall contact and structural preservation. In the mesial canals, increasing taper significantly influenced the percentage of unprepared surface and the amount of dentin removed, whereas in distal canals, taper significantly affected dentin removal but did not significantly reduce the extent of unprepared canal surface. The null hypothesis was therefore partially rejected. Clinically, this suggests that increasing taper beyond 0.05 in mesial roots leads to a meaningful improvement in shaping coverage; however, this benefit is accompanied by considerable dentin loss, especially in anatomically critical areas. An important factor underlying the present findings is the marked difference in coronal diameter (D16) between instruments with the smallest and largest tapers. In mesial roots of mandibular molars, dentin preservation in the coronal and middle thirds is strongly influenced by instrument dimensions. In this study, the D16 increased from approximately 0.73 mm with the 25/0.03 instrument to approximately 1.05 mm with the 25/.08v instrument, which helps explain the greater dentin removal observed in these regions, particularly on the distal aspect of the mesial canals, underscoring that taper-related dentin loss is largely governed by coronal instrument size rather than apical enlargement alone.

The large amount of unprepared surface seen after using smaller tapers is consistent with earlier micro-CT studies, which have shown that a significant part of the canal wall often remains untouched after mechanical preparation, even when modern NiTi systems are used [23–26]. Studies of mesial roots in mandibular molars have described complex shapes, such as isthmuses, flattened bucco-lingual areas, and deep irregularities that instruments often fail to reach [4]. The present results support these findings by showing that, with 25/0.03 and 25/0.05 instruments, more than one third of the original canal surface in mesial canals can remain unprepared (Figs. 1a and 2a; Table 1), confirming previous reports that mechanical enlargement alone has important limitations [27–29]. Although a reduction in the percentage of unprepared canal surface does not necessarily translate into improved clinical outcomes or complete microbial elimination, the interpretation of this parameter remains challenging. Importantly, no established threshold currently exists to define how much reduction in unprepared surface would be required to produce meaningful benefits. Nevertheless, reductions in untouched areas may facilitate irrigant penetration, fluid exchange, and chemical debridement in anatomically complex regions of the canal system, such as fins, recesses, and isthmuses, where mechanical instrumentation is inherently limited.

It should also be considered that, in micro-CT analyses, static voxels represent areas where no detectable dentin removal has occurred within the spatial resolution limits of the imaging system [30]. Therefore, regions classified as unprepared do not necessarily indicate a complete absence of instrument contact; rather, they reflect areas where any interaction with the instrument did not result in measurable structural alteration. Consequently, some canal surfaces may have been contacted or lightly touched during instrumentation without producing sufficient material removal to be detected by the imaging method. From a clinical perspective, these regions may still benefit from chemical debridement, as irrigant penetration, fluid exchange, and activation protocols can contribute to biofilm disruption and debris removal in areas where mechanical instrumentation is limited [4].

In agreement with previous studies [19, 31, 32], increasing taper improved contact with the canal walls in mesial canals, as shown by the significant reduction in unprepared areas when the 25/0.06 and 25/.08v instruments were used (Figs. 1a and 2a; Table 1). Earlier research has also shown that larger tapers help instruments to contact wider areas of the canal and shape extensions of oval canals more effectively [33–36]. However, the amount of dentin removed in the present study, especially the increase of about 252% with 25/0.06 and about 350% with 25/.08v compared with 25/0.03 (Fig. 2a; Table 1), raises important concerns about the biological impact of large-taper preparations. Such excessive dentin removal may predispose teeth to strip perforation and increase susceptibility to vertical root fracture, particularly in mesial roots of mandibular molars [15]. Other studies have also reported that dentin removal increases rapidly as taper increases, and have warned that more aggressive tapers can weaken the root structure and increase the risk of vertical root fracture [23, 37].

The present findings are consistent with previous micro-CT investigations that evaluated the relationship between instrumentation strategies and dentin preservation in mandibular molars. Silva et al. [38] evaluated the shaping performance of TruNatomy and ProTaper Gold systems in mandibular molars and reported similar percentages of unprepared canal walls and remaining dentin thickness between the systems, despite differences in taper and design. The authors also observed that ProTaper Gold removed significantly more dentin at the coronal level of the mesial roots, whereas TruNatomy tended to preserve dentin more effectively without compromising shaping ability. These observations are in line with the present results, which demonstrated that increased taper was associated with progressively greater dentin removal, particularly in the coronal and middle thirds of mesial roots. Importantly, our stepwise analysis further showed that increases in taper do not necessarily translate into proportional improvements in canal wall contact, reinforcing the concept that more aggressive tapers may increase structural sacrifice without providing substantial gains in shaping efficiency.

Similarly, the recent study by Alovisi et al. [37] combined micro-CT analysis with finite element modelling to evaluate the effect of different tapers on dentin preservation and biomechanical behavior of mandibular molar mesial roots. Their findings indicated that systems with reduced taper preserved significantly more cervical dentin and exhibited improved centering ability, particularly in the coronal and middle thirds, while larger tapers resulted in greater dentin removal in the danger zone. These findings closely align with the directional dentin reduction pattern observed in the present study, where dentin removal was consistently greater on the furcal aspect of mesial roots. Together, these results reinforce the importance of cautious taper selection in anatomically vulnerable areas, where excessive enlargement may compromise structural integrity without clear biomechanical benefits.

Earlier micro-CT investigations also highlighted the influence of canal anatomy on shaping outcomes. Zhao et al. [39] evaluated the shaping behavior of ProTaper Universal, ProTaper Next, and WaveOne systems in mandibular molars and reported that distal canals exhibited a significantly greater proportion of unprepared surfaces than mesial canals, despite instrumentation. Their results demonstrated that canal morphology plays a critical role in shaping outcomes, regardless of the instrumentation system used. The present study corroborates this concept by showing that taper enlargement significantly reduced unprepared surfaces in mesial canals but had limited influence in distal canals. This difference likely reflects the more flattened and irregular morphology of mesial canals compared with the typically rounder distal canals, which alters how instruments interact with canal walls.

It is important to note, however, that most previous studies have compared different instrumentation systems, in which taper, alloy, cross-sectional design, and kinematics vary simultaneously, making it difficult to isolate the individual contribution of each factor to shaping outcomes. In contrast, the present study was specifically designed to evaluate the isolated effect of taper enlargement while maintaining a constant apical diameter, using a stepwise experimental design within the same specimens. This approach minimizes anatomical variability and allows a more precise assessment of the structural trade-off between shaping efficiency and dentin preservation. Taken together, these findings support the notion that root canal shaping involves a delicate balance between improving mechanical contact and preserving dentin structure. By isolating instrument taper as the principal variable and analyzing its progressive effects through sequential micro-CT evaluation, the present study provides additional insight into how taper enlargement influences this balance in anatomically complex mandibular molars.

In the distal canals, the lack of a significant decrease in unprepared surface, compared with the mesial canals, highlights how root anatomy influences shaping results. Distal canals in mandibular molars are usually rounder and wider than mesial canals, and even though they were prepared to a larger apical size (40), increasing the taper from 0.03 to .06v did not lead to a statistically significant improvement in surface coverage (Figs. 1a and 2a; Table 1). This finding suggests that, in more circular canals, increasing taper adds little benefit in terms of wall contact, while still removing more dentin, which may not be necessary from a biological point of view.

The gradual decrease in unprepared areas in mesial canals is likely due to better contact between the instruments and the long, flattened canal walls as taper increases. Instruments with larger tapers shape the coronal and middle thirds more aggressively, which allows them to reach areas that thinner instruments cannot. However, this increased contact also explains the step-by-step and consistent reduction in dentin thickness seen on the furcal side of the mesial roots (Figs. 1b and 2b-d). The directional analysis showed that dentin reduction followed the pattern MB > ML > D at all levels, which can be explained by the typical anatomy of mandibular molars. The furcal sides of the mesiobuccal and mesiolingual canals usually have the thinnest dentin walls, known as “danger zones” [11–13]. Because larger-taper instruments tend to straighten inside the canal and remove more dentin from the inner, curved side, these thin areas are more affected [5, 14, 19]. The greater effect in the MB canal compared with the ML canal may be related to small, but common, differences in canal curvature and initial wall thickness. The fact that distal canals showed significant increases in dentin removal without a similar decrease in unprepared surface supports the idea that increasing taper can enlarge the canal and thin the walls without necessarily improving cleaning of complex areas. In practical terms, this means that increasing taper offers fewer benefits in canals that already have a more favorable, round shape.

Some studies have proposed threshold values for dentin thickness to safeguard the mechanical integrity of roots. For instance, Lim and Stock [40] suggested that dentin thickness values below 0.3 mm may compromise root strength, whereas other authors have proposed a threshold of 0.5 mm [41]. Although these thresholds remain somewhat arbitrary, the value of 0.5 mm has frequently been adopted in experimental studies to facilitate comparisons across investigations. In the present study, a similar threshold-based analysis was performed to better interpret the structural implications of taper enlargement. Notably, none of the specimens exhibited dentin thickness below 0.5 mm before preparation, and only one measurement below this threshold were detected after instrumentation (Table 1). However, a progressive increase in measurements within the 0.5–1.0 mm range was observed as taper increased, particularly on the mesial roots. This finding indicates that although taper enlargement did not reduce dentin thickness to levels considered critically thin, it did shift a considerable number of measurements toward a potentially vulnerable range, especially in the MB and ML canals. Previous studies have reported similar observations. For example, analyses of mandibular molars prepared with Reciproc R25 and XP-Endo Shaper instruments showed that reductions below the 0.5 mm threshold occurred primarily in cases where the original dentin thickness was already relatively thin (< 0.65 mm), suggesting that the preoperative anatomy plays a decisive role in determining structural outcomes after preparation [42]. Likewise, studies evaluating other instrumentation protocols in mandibular molars, such as ProTaper Next X2 and X3 or Hero 642 sizes 30/0.04 and 30/0.06, reported preoperative dentin thickness values greater than approximately 0.88 mm and similarly did not observe reductions below the critical threshold following preparation [11, 43]. Taken together, these findings reinforce the notion that the initial dentin thickness of the mesial roots of mandibular molars is a key determinant of the risk of reaching critical structural thresholds, often exerting a greater influence than the specific preparation protocol.

One noteworthy finding was that the 25/0.05 instrument did not significantly reduce the unprepared surface in mesial canals when compared with 25/0.03, even though it removed much more dentin (Fig. 2a; Table 1). This suggests that a small increase in taper mainly removes dentin from areas that were already in contact with the instrument, instead of reaching areas that were previously untouched. Only when the taper increased to 0.06 or .08v did a statistically and clinically meaningful reduction in unprepared surface occur (Fig. 2a; Table 1). This non-linear relationship between taper and shaping efficiency challenges the common idea that every increase in taper leads to a proportional improvement in canal wall contact. Similarly, in the distal canals, no significant improvement in surface preparation was seen even with the largest taper tested (Fig. 2a; Table 1). This highlights an important point that a greater taper is not always better and should be chosen carefully according to the root anatomy, rather than being used routinely in all canals.

A key strength of this study is the use of high-resolution micro-CT with stepwise enlargement and accurate matching of pre- and post-instrumentation scans. This approach allowed precise 3D measurement of unprepared surface, dentin removal, and changes in wall thickness. The sequential design in the same specimens reduced variability and isolated the true effect of taper. Including both mesial and distal roots also increased the clinical relevance of the findings. In addition, the use of appropriate statistical models strengthened the reliability of the results. However, some limitations should be acknowledged. The present study was not designed to isolate the individual effects of kinematics, metallurgy, or cross-sectional design. Although a consistent instrumentation strategy was adopted whenever possible, the use of reciprocating instruments at the largest tapers (25/.08v and 40/.06v) was dictated by instrument availability, as equivalent rotary instruments with the same apical size and taper from the same manufacturer are not currently available. Consequently, the findings should be interpreted as taper-related and descriptive, rather than as a direct comparison of kinematic or metallurgical features. Another limitation of this study is that instrumentation was performed using a standardized pecking motion in order to ensure experimental reproducibility. However, in clinical practice, operator-dependent techniques such as brushing or directional instrumentation may influence the extent of canal wall contact and shaping efficiency, and these variables were not specifically evaluated herein. Finally, the study focused on structural outcomes and did not directly assess microbial reduction, irrigation effectiveness, or long-term resistance to fracture.

From a clinical point of view, these findings show that increasing taper is a double-edged sword. It can improve shaping in flat, complex canals, such as the mesial canals of mandibular molars, but it also reduces dentin in areas that are already vulnerable. In distal canals and in more round canal shapes, a larger taper may not be needed and can even be harmful, because it removes more dentin without providing much extra wall contact. These results support a more conservative, anatomy-based choice of taper, favouring smaller tapers whenever proper shaping and irrigation can still be achieved. Future studies should link taper-related dentin loss with the risk of root fracture and with the ability of irrigants to penetrate the canal and remove biofilm. Combining micro-CT data with computational fluid dynamics and finite element analysis could further clarify the functional trade-offs between shaping efficiency, irrigation effectiveness, and structural preservation. In addition, studies evaluating newer heat-treated alloys, adaptive or expandable instruments, and different kinematics may help identify approaches that improve cleaning while reducing unnecessary dentin loss.

## Conclusions

Increasing instrument taper consistently increased dentin removal and reduced dentin thickness in both mesial and distal roots, with a more pronounced and predictable reduction in unprepared areas in the mesial canals. Only the largest tapers (0.06 and 0.08v) significantly decreased unprepared surfaces in mesial canals, while smaller tapers better preserved canal walls. In distal canals, unprepared areas were less sensitive to taper, although dentin removal still increased with larger instruments. Overall, dentin reduction followed the pattern MB > ML > D across all levels, confirming that larger tapers improve shaping efficiency at the expense of greater dentin sacrifice.

## Data Availability

No datasets were generated or analysed during the current study.
